# Distal renal tubular acidosis and severe hypokalemia: a case report and review of the literature

**DOI:** 10.1186/s13256-019-2056-1

**Published:** 2019-04-26

**Authors:** George Vasquez-Rios, David John Westrich, Isaac Philip, John C. Edwards, Stephanie Shieh

**Affiliations:** 1 Department of Internal Medicine, St. Louis University School of Medicine, St. Louis, MO USA; 2 St. Louis University School of Medicine, St. Louis, MO USA; 3 Nephrology Division, Department of Internal Medicine, St. Louis University, St. Louis, MO USA; 4 Division of Nephrology, VA St. Louis Health Care System, St. Louis, MO USA

**Keywords:** Potassium balance, Renal tubular acidosis, Autoimmune diseases, Sicca syndrome, Metabolic acidosis, Chronic kidney disease, Case report

## Abstract

**Background:**

Distal renal tubular acidosis is a relatively infrequent condition with complex pathophysiology that can present with life-threatening electrolyte abnormalities.

**Case presentation:**

We describe a case of a 57-year-old Caucasian woman with previous episodes of hypokalemia, severe muscle weakness, and fatigue. Upon further questioning, symptoms of dry eye and dry mouth became evident. Initial evaluation revealed hyperchloremic metabolic acidosis, severe hypokalemia, persistent alkaline urine, and a positive urinary anion gap, suggestive of distal renal tubular acidosis. Additional laboratory workup and renal biopsy led to the diagnosis of primary Sjögren’s syndrome with associated acute tubulointerstitial nephritis. After potassium and bicarbonate supplementation, immunomodulatory therapy with hydroxychloroquine, azathioprine, and prednisone was started. Nonetheless, her renal function failed to improve and remained steady with an estimated glomerular filtration rate of 42 ml/min/1.73 m^2^. The literature on this topic was reviewed.

**Conclusions:**

Cases of renal tubular acidosis should be carefully evaluated to prevent adverse complications, uncover a potentially treatable condition, and prevent the progression to chronic kidney disease. Repeated episodes of unexplained hypokalemia could be an important clue for diagnosis.

## Background

Distal renal tubular acidosis (dRTA) is characterized by a failure to acidify the urine in the distal parts of the nephron [1, 2]. Frequently, patients present with minimal or no symptoms, which can lead to a delay in diagnosis. Progressively, it can lead to marked acid-base abnormalities, including hyperchloremic metabolic acidosis and severe hypokalemia, which can be fatal. In children, dRTA is usually associated with a genetic defect or anatomic abnormality of the urinary system [3]. In contrast, dRTA in adults is frequently related to acquired conditions such as infections, drugs, and autoimmune diseases. We describe a case of a woman with multiple episodes of severe hypokalemia and weakness as the main reason for admission.

## Case presentation

A 57-year-old Caucasian woman presented to our institution with severe muscle weakness, fatigue, and weight loss for the past 2 years. Her medical history included well-controlled migraines and depression, which were treated with sumatriptan and citalopram, respectively. In addition, she had chronic hypokalemia leading to multiple visits to the emergency department for muscle weakness. These episodes were treated with potassium supplementation, with only transient improvement. She denied smoking, drinking alcohol, or using recreational drugs. On further questioning, she complained about dry eyes and dry mouth for the past 5 months. Also, she mentioned unintentional weight loss of 8 pounds during the same time. Upon examination, her vital signs were within acceptable limits. She was cachectic, with marked temporal wasting, dry mouth, and poor dentition. No thrush was noticed. Her cardiopulmonary evaluation was unremarkable, and no organomegaly was palpated. Her neurological examination revealed decreased muscle strength in upper and lower extremities, both proximally and distally. Furthermore, her tendon reflexes were decreased throughout. However, her sensory and vibratory function was intact.

### Diagnostic methods

Biochemical studies showed hyperchloremia (122 mEq/L), nonanion gap (non-AG) metabolic acidosis (HCO_3_^−^, 16 mEq/L; AG corrected for albumin, 7.8 mEq/L), and severe hypokalemia (2.5 mEq/L). In addition, her serum creatinine (Cr) was 1.3 mg/dl (estimated glomerular filtration rate [eGFR], 42 ml/min/1.73 m^2^ per the Modification of Diet in Renal Disease formula [MDRD]), and her blood urea nitrogen was 16 mg/dl. The remaining electrolytes, including calcium, magnesium, and phosphorus, were within normal limits. Her arterial blood gas showed pH 7.29, partial pressure of carbon dioxide 26 mmHg, and partial pressure of oxygen 134 mmHg. Her urine biochemistry revealed specific gravity 1.004, urine osmolality 175 mOsm/L, and pH 7.0. On further evaluation, the patient had a high urine anion gap (UAG) of + 23 and an inappropriately high potassium-to-creatinine ratio (K/Cr) of 3.9 mEq/mg. Repeated urine studies showed persistent alkaline urine (pH range, 6.5–7) with no evidence of glycosuria or phosphaturia. These findings were concerning for dRTA complicated with severe symptomatic hypokalemia. Additionally, her urine sediment was notable for sterile pyuria, as well as the presence of eosinophils, which suggested an ongoing tubulointerstitial process.

**Fig. 1:**
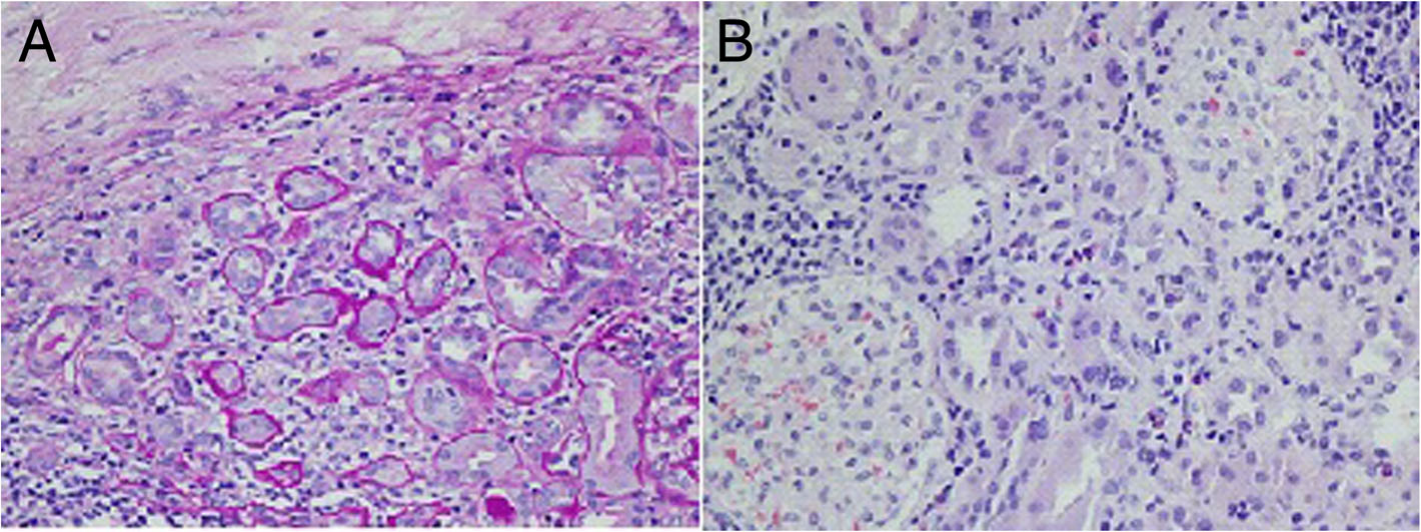
**a** Periodic acid-Schiff stain showing tubular atrophy, interstitial fibrosis, and inflammatory infiltrate in the glomeruli. **b** H&E stain showing eosinophil infiltrate in the interstitium, tubular atrophy, and intact glomeruli

She had mild polyclonal gammopathy with predominance of immunoglobulin G (IgG) antibodies and undetectable IgG4 levels. Furthermore, antinuclear antibody titers (1:1280, speckled pattern), antibodies against Sjögren’s syndrome antigen A (116.4; reference, 0–19.9), and antibodies against Sjögren’s syndrome antigen B (58.3; reference, 19.9) were very high, suggesting Sjögren’s syndrome (SS). The patient had no antibodies against salivary protein 1 or parotid-specific proteins. Antibodies against carbonic anhydrase (CA) type VI were negative as well. A renal biopsy was conducted, which revealed acute tubulointerstitial nephritis (TIN) with abundant eosinophils and significant lymphocytic and plasmatic cell infiltration (Fig. 1a and b). We concluded that our patient had primary SS with acute TIN.

### Treatment and outcome

The patient received aggressive therapy with potassium chloride (180 mEq/day), sodium bicarbonate (1960 mg/day), and amiloride (10 mg/day). In addition, she was treated with immunomodulatory therapy, including hydroxychloroquine (HCQ; 300 mg/day), azathioprine (50–100 mg/day), and a taper of prednisone. The patient tolerated the therapy and was reevaluated as an outpatient. After 2 weeks of inpatient treatment, her potassium level remained stable (3–3.5 mEq/dl), and she was minimally symptomatic. She was discharged with close follow-up.

Her strength and weight increased over the following 5 months. However, her renal function remained decreased with a serum Cr of 1.3–1.5 mg/dl, mild hypokalemia (K^+^, 3.1–3.4 mEq/dl), and mild metabolic acidosis (HCO_3_^−^, 20 mEq/L), punctuated by recurrent episodes of severe hypokalemia and acidosis when she was unable to maintain the high-dose potassium and bicarbonate supplementation. Figure 3 shows the trajectories of serum potassium levels and renal function as well as the influence of medical therapy during the clinical course of the patient. Her clinical course was affected by her intermittent compliance with prednisone owing to its side effects, most importantly edema and lipodystrophy. She developed chronic kidney disease (CKD) in the setting of TIN.

## Discussion

This case report describes a patient with recurrent hypokalemia. Her clinical manifestations included severe muscle weakness in upper and lower extremities, weight loss, and marked acid-base disorders such as hyperchloremic metabolic acidosis and severe hypokalemia. Her urine studies showed persistent alkaline urine, a positive UAG, and urinary eosinophils, suggestive of dRTA and an underlying nephritis. On further evaluation, the patient was diagnosed with SS and acute TIN. She was treated with prednisone, azathioprine, and HCQ in addition to potassium and bicarbonate supplementation, which helped to maintain acceptable serum potassium levels. However, the patient’s renal function failed to improve and transitioned to CKD. This is a unique case of repeated episodes of hypokalemia related to an immunological insult that ultimately induced progressive renal failure. Despite combined immunomodulatory therapy, the patient’s outcomes were marginal. This case provides a physiological perspective on a poorly understood condition: TIN and SS.

Distal RTA, also known as type 1 RTA or classic RTA, is a complex entity characterized by an inability to acidify the urine; a process that occurs in the distal parts of the nephron, including the connecting tubule and the collecting duct [4]. Little is known about the prevalence of this condition in the general population. In Thailand, one study revealed that the prevalence of dRTA was 2.8%, concerning for an endemic form of this disease [5]. However, the prevalence of dRTA can be as high as 22–25% in specific populations, such as in patients with osteopenia and SS [6–9]. In children, dRTA may result from genetic mutations that affect the normal acidification system, including the genes *ATPV0A4* and *ATP6V1B1*, which encode the subunits A4 and B1 of the proton ATPase (H^+^-ATPase), respectively, or defects in the gene *SCL4A1* that encodes anion exchange proteins [10–12]. In addition, dRTA can result from an abnormality of the ureteropelvic system, such as medullary sponge kidney, obstructive uropathies, or pyelonephritis. Conversely, dRTA is frequently associated with autoimmune diseases, medications, and parathyroid disorders in the adult population [1, 2].

Acid secretion by the kidney can be conceptualized as having two components: (1) reclamation, which involves the reabsorption of the filtered bicarbonate load, a mechanism that is preserved in patients with dRTA; and (2) regeneration, which occurs in the distal parts of the nephron to further excrete the excess of nonvolatile acids generated from the diet. Urinary acidification and regulation of acid-base balance result from the integrated function of the collecting duct, where the transport pathways for sodium, potassium, and protons (H^+^) are tightly intertwined. The cortical collecting duct is comprised of three distinct cell types that conduct very different transepithelial transport activities: principal cells, α-intercalated cells (A-intercalated cells), and β-intercalated cells (B-intercalated cells). The principal cells are the site of electrogenic sodium reabsorption via the apical epithelial sodium channel and the basolateral sodium-potassium ATPase (Na^+^/K^+^-ATPase). Transport through this pathway is upregulated by aldosterone and can be limited by apical sodium delivery and urine flow. The net effect of this transport is the generation of a lumen-negative transepithelial electrical potential. Principal cells also express voltage-gated apical potassium channels that, also in concert with the basolateral Na^+^/K^+^-ATPase, support K^+^ secretion. Hence, the lumen-negative electrical potential generated by sodium reabsorption is a key driving force for K^+^ secretion to the urine.

**Fig. 2:**
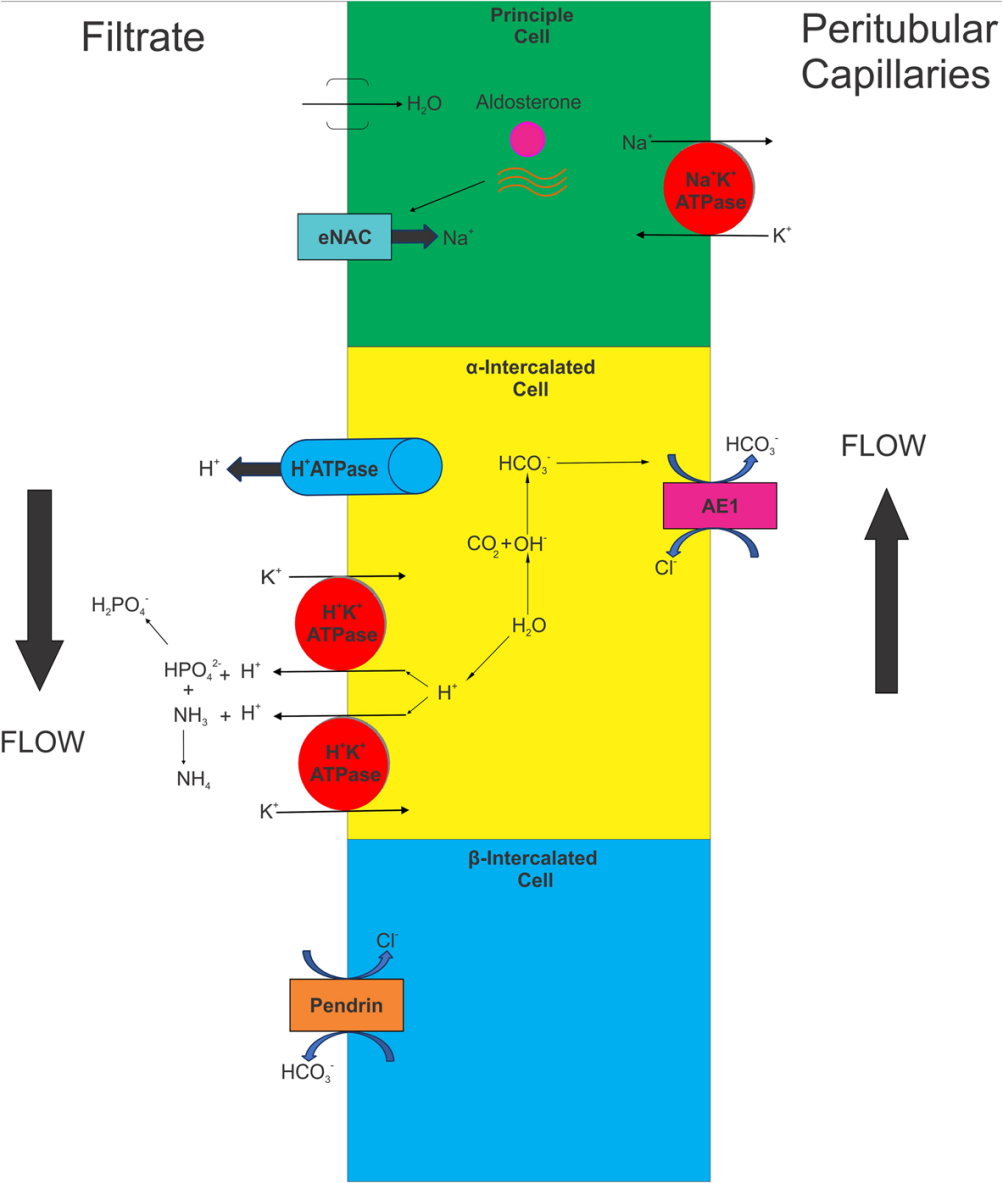
Main physiological functions of the principal cell, α-intercalated cell, and β-intercalated cell. *AE1* Anion exchanger 1, *eNAC* Epithelial sodium channel

A-intercalated cells are the site of electrogenic H^+^ secretion that is responsible for urinary acidification. These cells express high levels of intracellular CA type II, which generates carbonic acid (H_2_CO_3_) from carbon dioxide (CO_2_) and water. In the intracellular compartment, H_2_CO_3_ dissociates to H^+^ and a bicarbonate ion (HCO_3_^−^). The proton is secreted into the lumen across the apical membrane via the H^+^-ATPase, whereas HCO_3_^−^ exits across the basolateral membrane in exchange for chloride via the chloride-bicarbonate exchanger (AE1). Furthermore, chloride recycles in the basolateral aspect of the cell through a chloride channel. Thus, A-cells dissipate the lumen-negative potential generated by sodium reabsorption through the secretion of both H^+^ and K^+^, a unique feature of the distal regulation of acid load. B-intercalated cells are involved in HCO_3_^−^ secretion and K^+^ reabsorption and are not discussed further here. Figure 2 shows some of the main functions of the principal, A-intercalated, and B-intercalated cells.

The most common form of dRTA is due to selective failure of activity or expression of the H^+^-ATPase. The decreased transit through the proton pump inhibits urine acidification and reduces the electrical dissipation of the membrane potential. The latter has been suspected to be a driving force for K^+^ secretion and eventual potassium wasting in previous studies [13–17]. Norgett *et al.* [18] reproduced this hypothesis in knockout mice with deficient expression of the gene *ATP6V0A4* and found that these mice developed severe hyperchloremic metabolic acidosis, hypokalemia, and early nephrocalcinosis when challenged with acid load, features encountered in patients with severe dRTA. As hypokalemia progresses, storage tissues such as the skeletal muscle compensate by releasing K^+^ to the extracellular compartment, for which laboratory data may fail to uncover a K^+^ imbalance. However, a serum K^+^ < 3 mEq/L is related to a total body deficit of > 200 mEq, which varies with weight [19]. Patients with SS and dRTA can present life-threatening complications owing to massive intracellular potassium depletion, including rhabdomyolysis, respiratory paralysis, or malignant arrhythmias [13, 14, 20, 21].

The exact mechanism by which SS can induce dRTA remains unclear. However, previous studies evidenced a downregulated expression of the vacuolar H^+^-ATPase in the A-intercalated cells in patients with SS with concomitant underexpression of AE1 (pendrin) in the B-intercalated cells. It is hypothesized that the reduced secretion of H^+^ is the primary dysfunction in SS, whereas the underregulation of pendrin is a compensation to suppress HCO_3_^−^ secretion and prevent further acidosis [13, 14, 17]. Despite the presence of H^+^-ATPase and AE1 in other parts of the nephron, patients with SS present selective underexpression of these proteins in the collecting duct. Furthermore, patients with SS can also develop antibodies against components of the cellular membrane or intracellular proteins. Devuyst *et al.* [15] found IgG autoantibodies in a patient with SS that reacted against A-intercalated cells of a human control kidney. However, the target protein was not identified. Additional studies have identified antibodies against enzymes involved in the acid excretion system and bicarbonate generation, including CA type II, CA type IV, and CA type VI [22–26]. It is not clear whether these antibodies are part of the pathogenesis of SS or if they result from the exposure of intracellular epitopes to the immune system during tubular damage [13, 14, 22, 27]. Our patient tested negative for CA-VI specific antibodies. However, a prospective evaluation is needed because these antibodies may appear over the course of disease.

**Fig. 3:**
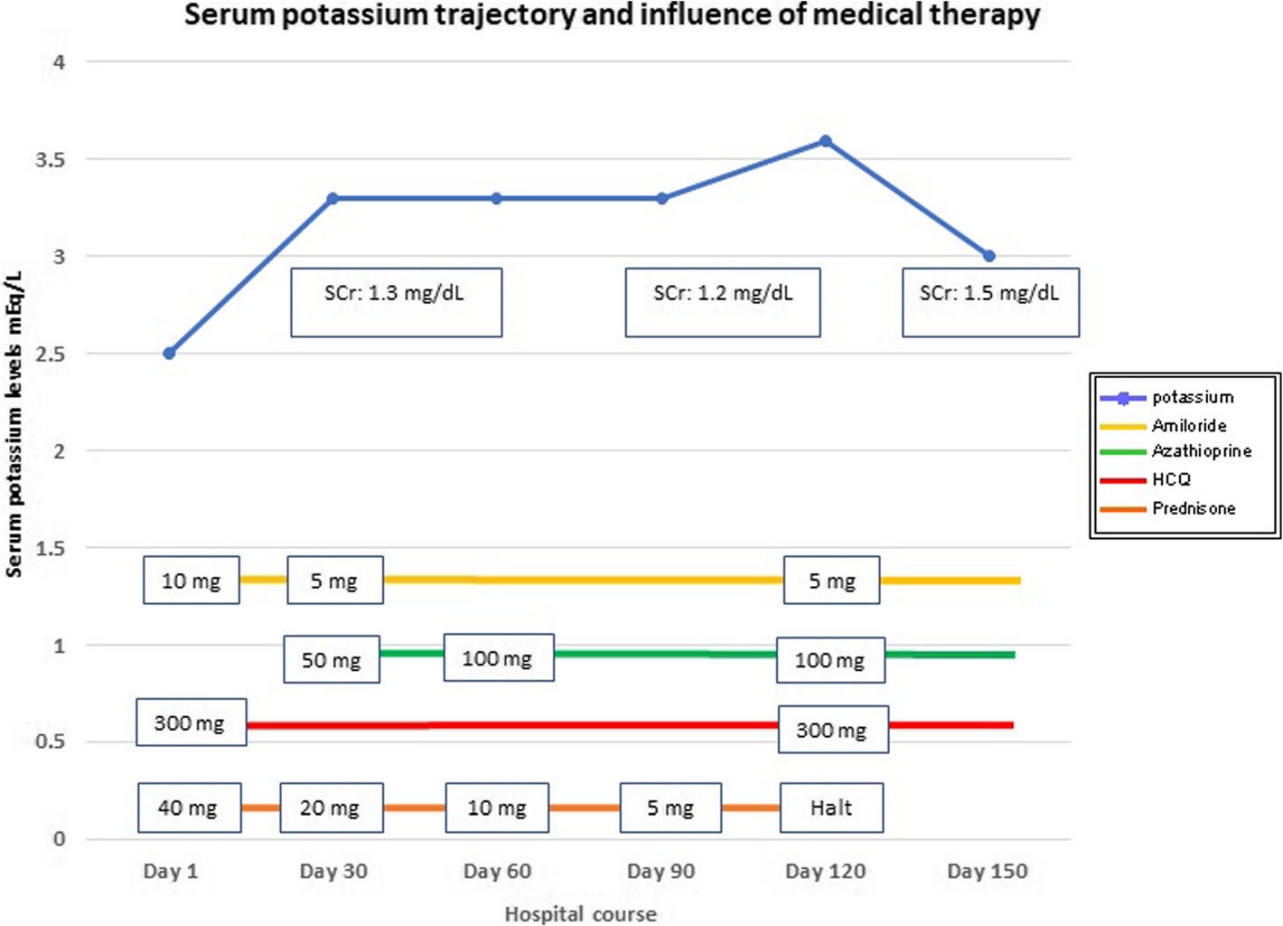
Serum potassium levels, medical therapy implemented, and clinical course of the patient

Our patient was treated with K^+^ and sodium bicarbonate supplementation to correct her acid-base imbalance. She also received amiloride as recent literature suggests that patients with severe disease may respond to this therapy [28]. Amiloride inhibits electrogenic sodium transport through the epithelial sodium channel in the principal cells and thus decreases the driving forces for electrogenic potassium secretion [28]. On follow-up, she maintained mild hypokalemia (K^+^, 3.1–3.4 mEq/L) as well as mild metabolic acidosis (HCO_3_^−^, 19–23 mmol/L). No further improvement was seen in these clinical parameters after the introduction of immunomodulatory therapy. Discontinuation of prednisone was related to worsening renal function as described in Fig. 3.

Up to 71% of the patients with SS and renal involvement may develop acute or chronic TIN, whereas cryoglobulinemic glomerulonephritis and focal segmental glomerulosclerosis are infrequent, accounting for < 5% of the cases [29]. Our patient presented an acute form of TIN with significant eosinophil/plasmatic cell infiltration and minimal tubular atrophy, which has been described elsewhere [30]. Figures 1 and 2 present the patient’s renal biopsy with characteristic findings described in SS. Some studies have reported specific histological findings in patients with SS and TIN, including lymphocytic infiltrate with predominance of T-helper cell populations, most prominently the Th-17 subpopulation [31]. These findings have also been noted in biopsy samples of the salivary gland of patients with SS, suggesting that blunting the primary immune response affecting salivary glands could also attenuate the inflammation in the kidneys. Thus, several reports extrapolate the therapy for patients with SS without renal disease to those with SS and renal involvement. To the best of our knowledge, very few studies have evaluated the response to immunomodulatory therapy in biopsy-proven TIN secondary to SS [29, 30]. Evans *et al.* [30] included 12 patients treated with a course of prednisone associated with mycophenolate mofetil (11 of 12) or azathioprine (1 of 12) to effectively affect both B-cell and T-cell populations. Interestingly, patients presented a significant response evidenced in their serum Cr levels and eGFR. Maripuri *et al*. [29] demonstrated that a mixed population including patients with SS and membranous proliferative glomerulonephritis would effectively respond to HCQ, cyclophosphamide, and rituximab. However, such therapy is difficult to reproduce in clinical practice, based on the low power of the study and the clinical characteristics of the participants.

While the immunomodulatory effect of HCQ relays in suppressing Toll-like receptors in a wide range of cells, azathioprine is an antimetabolite that inhibits synthesis of DNA, RNA, and proteins predominantly in T cells and B cells. Although both medications have been used for the management of this condition, outcomes have been variable in the literature. After 5 months of follow-up, our patient’s renal function was steady with an eGFR of 38–42 ml/min/1.73m^2^ (MDRD formula). She was dependent on high-dose potassium and bicarbonate supplementation to maintain her electrolyte homeostasis, which suggests that tubulointerstitial injuries in SS can have minimal response to standard immunomodulatory therapy. This group of patients benefits from follow-up and further management to prevent progression of CKD to end-stage renal disease [32].

## Conclusions

This case highlights the importance of an early detection and physiology-based approach to dRTA. Accurate characterization of this condition is pivotal to uncover an underlying disease, tailor a specific therapy, and prevent further renal function decline. Episodes of symptomatic hypokalemia could be a clue for diagnosis and should be carefully addressed to prevent life-threatening complications. Evaluating the renal response to immunomodulatory therapy in SS is of paramount interest as it can lead to a progressive decline in renal function.

## Abbreviations

AG: Anion gap
CA: Carbonic anhydrase
CKD: Chronic kidney disease
Cr: Creatinine
dRTA: Distal renal tubular acidosis
eGFR: Estimated glomerular filtration rate
HCQ: Hydroxychloroquine
IgG: Immunoglobulin G
MDRD: Modification of Diet in Renal Disease
SS: Sjögren’s syndrome
TIN: Tubulointerstitial nephritis
UAG: Urine anion gap
